# Supplementary material for: Ultrasound neuromodulation hypotheses on the directionality of effects and community resource
Source: eLife. 2026 Oct 28;13:RP100827. doi: 10.7554/eLife.100827 (PMC13716386; doi:10.7554/eLife.100827)
Supplement: Supplementary file 1 [file elife-100827-mdarchecklist1.pdf]

MDAR Checklist for Authors

Materials Design Analysis Reporting (MDAR) checklist completed for eLife (Version of Record).

Manuscript: Caffaratti H, Slater B, Shaheen N, et al., Petkov CI. Hypotheses on the directionality of ultrasound neuromodulation effects and a community resource. eLife Reviewed Preprint 100827.

The MDAR framework sets out a minimum set of requirements for transparent reporting in the life sciences. For each item, the location of the information in the manuscript (section or figure legend) or the submission form is given, or the item is marked as not applicable. This paper is a meta-analysis of published human transcranial ultrasound stimulation (TUS) studies, a review, and an open data resource; no new experiments were performed.

Items highlighted in yellow need confirmation by the authors before submission.

Materials

Materials	Response / where provided in the manuscript (section, figure legend or submission form)	N/A
Newly created materials	No new physical materials were created. The newly created resource is a dataset (the inTUS database), described under Data availability below. Section: "How to use and contribute to the inTUS Resource"; Data availability statement.	
Antibodies	Not applicable: this is a meta-analysis of published studies and a review; no new materials of this type were generated or used.	✓
DNA and RNA sequences	Not applicable: this is a meta-analysis of published studies and a review; no new materials of this type were generated or used.	✓
Cell materials – Cell lines	Not applicable: this is a meta-analysis of published studies and a review; no new materials of this type were generated or used.	✓
Cell materials – Primary cultures	Not applicable: this is a meta-analysis of published studies and a review; no new materials of this type were generated or used.	✓
Experimental animals – Laboratory animals or model organisms	Not applicable: No animal experiments were conducted. Animal studies are discussed only as published literature (Introduction; Part II).	✓
Experimental animals – Animals observed in or captured from the field	Not applicable: this is a meta-analysis of published studies and a review; no new materials of this type were generated or used.	✓
Plants and microbes – Plants	Not applicable: this is a meta-analysis of published studies and a review; no new materials of this type were generated or used.	✓
Plants and microbes – Microbes	Not applicable: this is a meta-analysis of published studies and a review; no new materials of this type were generated or used.	✓
Human research participants	No participants were recruited. The meta-analysis uses study-level parameters and outcomes extracted from published studies of healthy human participants (n = 47 studies, 52 experiments). Individual participant demographics (age, sex, gender, ethnicity) were not collected. Section: "Meta-analysis inclusion criteria and analysis approach"; Tables 1 and 2.	

Design

Design	Response / where provided in the manuscript (section, figure legend or submission form)	N/A
Study protocol	The meta-analysis was not pre-registered (e.g., no PROSPERO registration). This is not a clinical trial. The search and inclusion procedure follows PRISMA and is described in "Meta-analysis inclusion criteria and analysis approach" and Figure 3.	
Laboratory protocol	Not applicable: No laboratory protocols. The analysis workflow (R markdown script) is openly available on OSF (https://osf.io/arqp8/), see Code availability.	✓
Experimental study design (statistics details)	Not applicable: No in vivo experiments were performed. For the meta-analysis:	
Sample size determination	No a priori sample size calculation; all studies meeting the inclusion criteria up to 1 January 2025 were included (inTUS_DATABASE_6-2024: 37 experiments; inTUS_DATABASE_1-2025: 52 experiments from 47 studies). Limited statistical power is acknowledged in "Meta-analysis limitations". Sections: "Meta-analysis inclusion criteria and analysis approach"; Results.	
Randomisation	Not applicable (no allocation of subjects or samples to groups).	✓
Blinding	Study selection and data extraction were performed by four authors (HC, NS, CP and MZ) without blinding; the directionality of effects was categorized using independently published characterizations wherever possible (Tables 1–2, rightmost column).	
Inclusion/exclusion criteria	Inclusion and exclusion criteria are stated in "Meta-analysis inclusion criteria and analysis approach" and Figure 3 (PRISMA flow diagram): low-intensity TUS in healthy human participants, physiological measurement with clear directionality of effect, peer-reviewed English-language articles before 1 January 2025; moderate/high-intensity (BBB opening, ablation) studies and studies lacking required parameters were excluded.	
Sample definition and in-laboratory replication	The unit of analysis is the experiment: when a study reported multiple complete experiments, each was entered separately; where separate experiments varied a parameter with the same result, the experiment with the strongest effect was entered. The analysis was repeated at two database evaluation periods (6-2024 and 1-2025). Section: "Meta-analysis inclusion criteria and analysis approach"; Results.	
Ethics – Studies involving human participants	No new data were collected from human participants; the work is a secondary analysis of published, study-level data, so ethics approval for secondary hypothesis testing was not required.	✓
Ethics – Studies involving experimental animals	Not applicable: No animal experiments were performed.	✓
Ethics – Studies involving specimen and field samples	Not applicable: No specimens or field samples were used.	✓
Dual Use Research of Concern (DURC)	Not applicable.	✓

Analysis

Analysis	Response / where provided in the manuscript (section, figure legend or submission form)	N/A
Attrition	Exclusion criteria were defined before data extraction (Methods; Figure 3). Of 960 records identified, 900 were removed before screening, 60 reports were assessed and 13 excluded, leaving 47 studies (52 experiments). Four diagnostic ultrasound studies were excluded because they did not report the parameters needed for the analysis (Gibson et al., 2018; Guerra et al., 2021; Hameroff et al., 2013; Schimek et al., 2020). Where ISPPA in the brain was not reported (14 of 37 experiments in 6-2024; 13 of the 15 added experiments in 1-2025), values were estimated assuming 70–75% skull attenuation and checked against k-plan simulations (~5% error). Sections: "Meta-analysis inclusion criteria and analysis approach"; "Meta-analysis limitations"; Figure 3.	
Statistics	Exploratory univariate logistic regression models (R/RStudio) tested Enhancement versus Suppression separately for ISPPA (brain), duty cycle, PRF, sonication duration and ISPTA, with mode of stimulation (online/offline) as a covariate; a single full-interaction model was not used because the sample size is insufficient. An exploratory multivariate Random Forest classification (scikit-learn; 200 × 5-fold cross-validation) was compared with a shuffled-label control, with permutation importance for each parameter. P values, means and standard deviations are reported in Results. Sections: "Meta-analysis inclusion criteria and analysis approach"; Results; Figures 5 and 6.	
Data availability	Newly created dataset: inTUS database tables (inTUS_DATABASE_6-2024.csv; inTUS_DATABASE_1-2025.csv) with a column description file, deposited on the Open Science Framework, https://osf.io/arqp8/ (folder "Caffaratti_et_al_inTUS_Resource"). License is CC-BY 4.0.<br>Reused data: TUS parameters and outcomes extracted from the published studies cited in Tables 1 and 2. Data availability statement; Tables 1–2.	
Code availability	New code: R markdown script TUS_Effects_1-2025_Ranalyses.Rmd that regenerates the statistics and figures, deposited at https://osf.io/arqp8/; example output at https://rpubs.com/BenSlaterNeuro/1268823; interactive app at https://benslaterneuro.shinyapps.io/Caffaratti_inTUS_Resource/.<br>Reused software: R and RStudio (Posit), scikit-learn (Pedregosa et al., 2011), k-plan (BrainBox Ltd; Jaros et al., 2020; Treeby & Cox, 2010). Sections: "How to use and contribute to the inTUS Resource"; appendix "Introduction to the Iowa-Newcastle (inTUS) Transcranial Ultrasound Stimulation Resource".	

Reporting

Reporting	Response / where provided in the manuscript (section, figure legend or submission form)	N/A
Adherence to community standards	The meta-analysis follows the PRISMA (Preferred Reporting Items for Systematic reviews and Meta-Analyses) guidelines; the PRISMA flow diagram is provided as Figure 3. TUS parameter reporting follows ITRUSST consortium	

Reporting	Response / where provided in the manuscript (section, figure legend or submission form)	N/A
	recommendations (Martin et al., 2024). Section: "Meta-analysis inclusion criteria and analysis approach"; Figure 3.	
