# Supplementary material for: Ultrasound neuromodulation hypotheses on the directionality of effects and community resource
Source: eLife. 2026 Oct 28;13:RP100827. doi: 10.7554/eLife.100827 (PMC13716386; doi:10.7554/eLife.100827)
Supplement: Supplementary file 2 [file elife-100827-supp1.docx]

**Introduction to the Iowa-Newcastle (inTUS)**

**Transcranial Ultrasound Stimulation Resource**

The inTUS Resource consists of the following resource items, linked to Caffaratti et al. ***Neuromodulation with Ultrasound: Hypotheses on the Directionality of Effects and a Community Resource.*** Please cite the latest eLife paper when using and referring to the inTUS resource: <https://elifesciences.org/reviewed-preprints/100827>.

The inTUS Resource documents can be found on the Laboratory of Comparative Neuropsychology data share on the Open Science Framework: <https://osf.io/arqp8/> under the folder Caffaratti_et_al_inTUS_Resource. If the location of the repository changes, a link to its location will be left here.

**Resource documents**

- **Database Tables**
  - inTUS_DATABASE_1-2025.csv
    - Certain key historic versions will be retained, e.g., the initial inTUS_DATABASE_6-2024.csv
  - InTUS_DATABASE_1-2025_description.pdf describes the columns in the database
- **R Markdown script** to regenerate the figures and statistics using R Studio, and example PDF/HTML output if not using R to more quickly visualize TUS parameter effects.
  - TUS_Effects_1-2025_Ranalyses.Rmd is the executable R markdown script, which executes the statistical models and figures. To run the script, install the freely available R Studio software (<https://posit.co/>). Run the script sections as described in the instructions at the top of the script (tested in R Studio version RStudio-2024.12.0-467).
  - InTUS_OUTPUT_1-2025.pdf is an example output file generated by the latest database, if the reader more quickly wants to see the latest output without having to run the R markdown script: [https://rpubs.com/BenSlaterNeuro/1268823](https://nam12.safelinks.protection.outlook.com/?url=https%3A%2F%2Frpubs.com%2FBenSlaterNeuro%2F1268823&data=05%7C02%7Cchristopher-petkov%40uiowa.edu%7Cba105f55a80e4d129d8f08dd45d6a652%7C1bc445959aba4fc3b8ec7b94a5586fdc%7C1%7C0%7C638743510445387223%7CUnknown%7CTWFpbGZsb3d8eyJFbXB0eU1hcGkiOnRydWUsIlYiOiIwLjAuMDAwMCIsIlAiOiJXaW4zMiIsIkFOIjoiTWFpbCIsIldUIjoyfQ%3D%3D%7C0%7C%7C%7C&sdata=8vID%2FPfBFrBNLZFj0DwDerM9WKCswl6%2Bhx0HBr3VI3E%3D&reserved=0)
  - Data visualization online app to visualize the TUS parameters data: <https://benslaterneuro.shinyapps.io/Caffaratti_inTUS_Resource/>
- **To contribute to the database please use the Qualtrics Survey**
  - **Qualtrics Survey** to submit your paper and data so that it can be considered for addition to the database. Please use this link: <https://uiowa.qualtrics.com/jfe/form/SV_4VOvb0fdwvACDkO>

**Using the resource**

To regenerate the manuscript figures with the latest tables, download the latest *database table* and *R_script*. Run the script in R or R Studio making sure it can access the inTUS database you are interested in (e.g., that the database is visible in the R Studio working directory).

**Contributing to the resource**

Please use the Qualtrics Link and Form to submit your preprint or published paper data. These submissions will be checked and once verified will be added to the database: <https://uiowa.qualtrics.com/jfe/form/SV_4VOvb0fdwvACDkO>

**How the resource is curated**

To ensure that the database has TUS researcher checked information, we have established the Qualtrics form that can be used to submit a preprint or publication citation and associated TUS values and outcomes. The human TUS expert factor ensures that a skilled researcher checks the submitted data that could be included in the database. Please note the inclusionary and exclusionary criteria in the paper, which may change with future database expansions. We are exploring the possibility of a collaboration with the ITRUSST consortium to sustain the resource and will update the OSF link if the location of the inTUS resource changes.

**Only healthy humans?**

This resource was initially established with healthy human participant low-intensity TUS studies. We will continue to expand the inTUS resource with the TUS community’s help and input. We are open to the resource being extended to human patients and nonhuman animals of different species. Please use the Qualtrics Form if you’re interested in helping or have suggestions about the resource.
