# Supplementary material for: Ultrasound neuromodulation hypotheses on the directionality of effects and community resource
Source: eLife. 2026 Oct 28;13:RP100827. doi: 10.7554/eLife.100827 (PMC13716386; doi:10.7554/eLife.100827)
Supplement: Peer review history

# Editor report: eLife Assessment

**DOI:** 10.7554/eLife.100827.4.sa0

**Authors:** Maria Chait

**Affiliations:** University College London United Kingdom

**Keywords:** Convincing, Important

This paper is an **important** overview of the currently published literature on low-intensity focused ultrasound stimulation (TUS) in humans, providing a meta-analysis of this literature that explores which stimulation parameters might predict the directionality of the physiological stimulation effects. The overall synthesis is **convincing**. The database proposed by the paper has the potential to become a key community resource if carefully curated and developed.

# Referee report: Reviewer #1 (Public review)

**DOI:** 10.7554/eLife.100827.4.sa1

This paper is a relevant overview of the currently published literature on low-intensity focused ultrasound stimulation (TUS) in humans, with a meta-analysis of this literature that explores which stimulation parameters might predict the directionality of the physiological stimulation effects.

The pool of papers to draw from is small, which is not surprising given the nascent technology. It seems, nevertheless, relevant to summarise the current field in the way done here, not least to mitigate and prevent some of the mistakes that other non-invasive brain stimulation techniques have suffered from, most notably the theory- and data free permutation of the parameter space.

A database summarising the literature and allowing for quantitative assessment of these studies is a key contribution of the paper. If curated well, it can become a valuable community resource.

Comments on revisions:

The paper is much improved. There remain a few caveats the authors may want to address.

I'm not going to dwell on this if the authors don't agree, but remain critical about the inclusion of TPS in the discussion. It's comparing apples and oranges, and unless there's a personal interest the authors have in TPS, it remains puzzling why it is included in the first place. As per my previous review, the literature on TPS, and especially the main example cited, has been highly criticised, including national patient and medical associations. A mere disclaimer that more work is needed isn't enough, in this reviewer's opinion - I simply don't understand why the authors go out on a limb here when the rest of the paper is done so well and thoroughly.

# Author comment: Author response

**DOI:** 10.7554/eLife.100827.4.sa2

**Authors:** Hugo Caffaratti, Ben Slater, Nour Shaheen, Ariane E Rhone, Ryan Calmus, Michael Kritikos, Sukhbinder Kumar, Brian Dlouhy, Hiroyuki Oya, Timothy D Griffiths, Aaron Boes, Nicholas Trapp, Marcus Kaiser, Jerome Sallet, Matthew I Banks, Matthew A Howard, Mario Zanaty, Chris Petkov

**Affiliations:** University of Iowa Hospitals and Clinics Iowa City United States; Newcastle University Newcastle United Kingdom; University of Iowa Hospitals and Clinics Iowa City United States; University of Iowa Iowa City United States; University of Iowa Hospitals and Clinics Iowa City United States; University of Iowa Hospitals and Clinics Iowa City United States; Newcastle University London United Kingdom; University of Iowa Hospitals and Clinics Iowa City United States; University of Iowa Iowa City United States; University of Newcastle Newcastle upon Tyne United Kingdom; University of Iowa Iowa City United States; University of Iowa Hospitals and Clinics Iowa City United States; University of Nottingham Nottingham United Kingdom; Inserm Bron France; University of Wisconsin-Madison Madison United States; University of Iowa Iowa City United States; University of Iowa Hospitals and Clinics Iowa City United States; University of Iowa Iowa City United States

The following is the authors’ response to the previous reviews

**Reviewer #1 (Public review):**

Summary:

This paper is a relevant overview of the currently published literature on lowintensity focused ultrasound stimulation (TUS) in humans, with a meta-analysis of this literature that explores which stimulation parameters might predict the directionality of the physiological stimulation effects.

The pool of papers to draw from is small, which is not surprising given the nascent technology. It seems nevertheless relevant to summarize the current field in the way done here, not least to mitigate and prevent some of the mistakes that other non-invasive brain stimulation techniques have suffered from, most notably the theory- and data-free permutation of the parameter space.

The meta-analysis concludes that there are, at best, weak trends toward specific parameters predicting the direction of the stimulation effects. The data have been incorporated into an open database that will ideally continue to be populated by the community and thereby become a helpful resource as the field moves forward.

Strengths:

The current state of human TUS is concisely and well summarized. The methods of the meta-analysis are appropriate. The database is a valuable resource.

We thank the reviewer for their positive assessment of the revised manuscript and the potential importance of the resource to the TUS community.

Suggestions:

The paper remains lengthy and somewhat unfocused, to the detriment of readability. One can understand that the authors wish to include as much information as possible, but this reviewer is sceptical that this will aid the use of the databank, or help broaden the readership. For one, there is a good chunk of repetition throughout. The intro is also somewhat oscillating between TMS, tDCS and TUS. While the former two help contextualizing the issue, it doesn't seem necessary. In the section on clinical applications of TUs and possible outcomes of TUS, there's an imbalance of the content across examples. That's in part because of the difference in knowledge base but some sections could probably be shortened, eg stroke. In any case, the authors may want to consider whether it is worth making some additional effort in pruning the paper

We thank the reviewer for these suggestions. We have checked for redundancy and that the clinical review section is more balanced, although some of the sections have more TUS studies than others, therefore some imbalance is unavoidable. As some examples, we have condensed the “Stroke and neuroprotection in brain injury” section (lines 624-647). This helps to improve the clarity and readability of the manuscript.

The terms or concept of enhancement and suppression warrant a clearer definition and usage. In most cases, the authors refer to E/S of neural activity. Perhaps using terms such as "neural enhancement" etc helps distinguish these from eg behavioural or clinical effects. Crucially, how one maps onto the other is not clear. But in any case, a clear statement that the changes outlined on lines 277ff do not

We thank the reviewer for this point and agree that it is important to distinguish neural E/S, as we had intended, from behavioral effects. In the first instance and in several places we add ‘neural’ before enhancement/suppression. Also see Lines 276-279: **“**Probable net neural enhancement versus suppression was characterised as follows. Note that our use of the terms enhancement and suppression refers exclusively to the increase or decrease of neural activity, respectively, as measured by, neurophysiological methods (EEG-ERPs, BOLD fMRI, etc.) and does not imply equivalent changes in behavioural responses**”**

Please see also lines 108-116.

Re tb-TUS (lines 382ff), it is worth acknowledging here that independent replication is very limited (eg Bao et al 2024; Fong et al bioRxiv 2024) and seems to indicate rather different effects

We have updated this section by referencing Bao et al. and Fong et al., as examples of the limited independent replication of tbTUS results. Please see lines 392-396. “However, independent replication of these findings remains limited. For example, Bao, found reduced motor cortex excitability – measured as decreased TMS-MEP amplitude in M1 -- that lasted up to 30 minutes post-sonication (Bao et al., 2024). Whereas Fong reported no significant effects between tbTUS and sham conditions in M1 excitability (Fong et al., 2024).”

The comparison with TPS is troublesome. For one, that original study was incredibly poorly controlled and designed. Cherry-picking individual (badly conducted) proof-of-principle studies doesn't seem a great way to go about as one can find a match for any desired use or outcome. Moreover, other than the concept of "pulsed" stimulation, it is not clear why that original study would motivate the use of TUS in the way the authors propose; both types of stimulation act in very different ways (if TPS "acts" at all). But surely the cited TPS study does not "demonstrate the capability for TUS for pre-operative cognitive mapping". As an aside, why the authors feel the need to state the "potential for TPS... to enhance cognitive function" is unclear, but it is certainly a non-sequitur. This review feels quite strongly that simplistic analogies such as the one here are unnecessary and misleading, and don't reflect the thoughtful discussion of the rest of the paper. In the other clinical examples, the authors build their suggestions on other TUS studies, which seems more sensible.

This is an excellent point, and we have removed that statement replacing it with: “However, TPS effects studies remain highly limited and would require further study and comparison to effects with other TUS protocols.”. Please see lines 561-562. We thank the reviewer for the supportive comments on the rest of the review.
